# The Role of Computed Tomography in the Diagnosis of Congenital Sensorineural Hearing Loss

**DOI:** 10.1055/s-0044-1786827

**Published:** 2024-06-07

**Authors:** Mauricio Buschle, Rogerio Hamerschmidt, Jorge Eduardo Fouto Matias, Otavio Pereira Lima Zanini, Luiz Otavio de Mattos Coelho, Jose Fernando Polanski

**Affiliations:** 1 Universidade Federal do Paraná, Curitiba, PR, Brazil; 2 Hospital Iguaçu, Curitiba, PR, Brazil; 3 Diagnóstico Avançado por Imagem – DAPI, Curitiba, PR, Brazil; 4 Faculdade Evangélica Mackenzie do Paraná, Curitiba, PR, Brazil

**Keywords:** sensorineural hearing loss, congenital, temporal bone, tomography

## Abstract

**Introduction** One of the paths in the investigation of congenital sensorineural hearing loss (CSNHL) is to try to characterize its etiology through the inner ear evaluation using high resolution computer tomography (CT) scans. With minor malformation, it is not always possible for a simple visual inspection to recognize if the structure in the inner ear is normal or not.

**Objective** To verify if measurements of the inner ear are predictive of sensorineural hearing loss (SNHL) and suggest cutoff points of size limits.

**Methods** Retrospective cross-sectional study of inner ear CT scan measurements of 214 patients, 50 with congenital SNHL (CSNHL) and 164 acquired SNHL (ASNHL) (control group).

**Results** In the CSNHL group, central bony island (CBI) were 0.48 mm smaller ( *p* < 0.001), cochlear nerve aperture was (CNA) 0.10 mm smaller ( *p* < 0.001), and cochlea height was (CH) 0.15 mm smaller ( *p* < 0.001). Vestibular aqueduct (VA) and cochlea width (CW) were similar between groups (0.70 vs 0.72, *p* = 0.19, and 7.20 vs 7.15 *p* = 0.23). The predictive cutoff points for CSNHL were CBI = 3.6 mm, CAN = 1.4 mm, CH = 3.4 mm, CW = 7.0 mm, and VA = 0.9 mm.

**Conclusion** Congenital sensorineural hearing loss determined a decrease in CBI, opening of the cochlear nerve (OCN), and CW. Thus, these measures, at the cutoff points indicated, should make us aware of the diagnosis of congenital hearing loss.

## Introduction

The incidence of neonatal hearing loss is 1.1/1,000 in the United States, and the prevalence of mild hearing impairment or worse is 3.1% among children and adolescents. 1 When not identified during childhood, Hearing loss may harm the development of speech and language, and school, social, and emotional performance.

To identify the etiology of sensorineural hearing loss (SNHL), genetic and serological tests, an investigation of autoimmune diseases, and imaging tests can be used. With minor malformation, it is not always possible for a simple visual inspection to recognize if the structure is normal or not. Thus, today, one of the paths in the investigation of congenital SNHL (CSNHL) is to try to characterize its etiology through measurements of the inner ear, in millimetric scales, using high resolution computed tomographic exams of the temporal bones. The precise identification of the inner ear malformations has a direct impact on the diagnosis, prognosis, and treatment of patients with CSNHL, and the study of the standardization of size normality of inner ear structures, such as the cochlea, semicircular canals, internal auditory canal, and VA, can, therefore, be useful in its diagnosis.

The present study aimed to assess whether measurements of the inner ear structures, such as the central bony island (CBI), cochlear nerve aperture (CNA), cochlea height (CH), cochlea width (CW), and vestibular aqueduct (VA) are predictive of CSNHL, and, secondly, to identify cutoff points in the measurements of the inner ear structures.

## Methods

The study was approved by the institution's research ethics committee under the number 2.107.295 and was conducted in accordance with the Declaration of Helsinki. It was a cross-sectional and retrospective study of patients who underwent hearing loss investigation in a tertiary referral center. The study included patients who underwent investigation of acquired sensorineural hearing loss (ASNHL group), who formed the control group, and patients with congenital sensorineural hearing loss (CSNHL group) and who underwent computed tomography study. Those with inconclusive or incomplete information in medical records or image reports were excluded. The criterion used to define congenital hearing loss was based on clinical records. We chose independent, unpaired analysis, since inner ear measurements do not change according to age or gender; and a control group with ASNHL, since inner ear structure does not change in ASNHL. The measures studied were the CBI, CNA, CH, CW, and VA. The measurements were taken from the right and left inner ears in patients of both genders, and the average between them was considered.

### Image Acquisition and Analysis

High-resolution multidetector computed tomography (MDCT) images with at least 64 channels were used, with a maximum slice thickness of 0.625 mm and a 512 × 512-pixel matrix, which generated unilateral volumetric images of the right and left ears in the axial plane that were further transferred to a workstation for postprocessing and analysis. The images were analyzed by an experienced head and neck radiologist. The measurements were taken using electronic tomography calipers in millimeters.

**Fig. 1:**
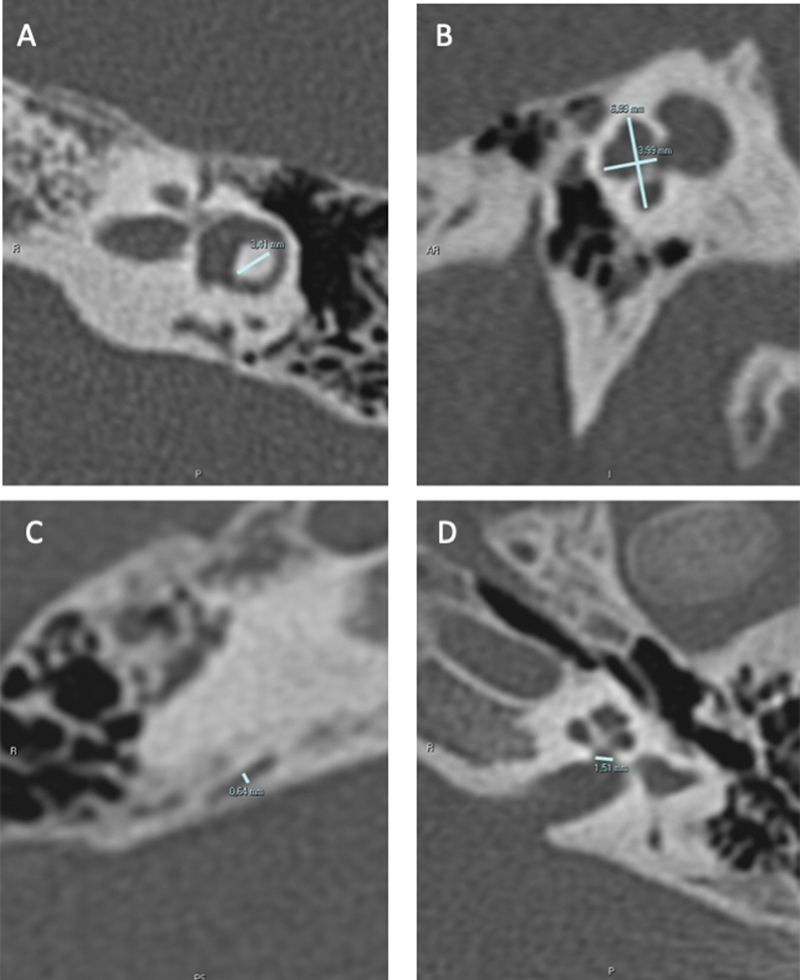
Inner ear measurements. A = central bony island; B = cochlear height and cochlea width; C = vestibular aqueduct; D = cochlear nerve aperture.

The width of the lateral semicircular canal CBI was measured in an axial section in a line connecting the apex of the canal with the communication means between the canal and the vestibule. The height and width of the basal turn of the cochlea were measured in a sagittal section at an oblique angle of the cut of greater height. Measurement of the VA was taken in the sagittal section, and the CW at the fundus of the internal auditory canal was measured in an axial plane. ( Fig. 1 )

### Audiologic Exams

Pure-tone audiometry was performed in an audiometric booth at frequencies of 250, 500, 1,000, 2,000, 3,000, 4,000, 6,000, and 8,000 Hz, and hearing loss (HL) was defined when > 25 dB at PTA (pure-tone average) (calculated using 500, 1,000, 2,000, 3,000 Hz - air conduction thresholds).

### Statistics

Measures of central tendency and dispersion are expressed as median and interquartile range values (median, IQR), given the asymmetrical distribution. The assumption of no normality was assessed using the Shapiro-Wilk test.

In the statistical analysis, the Mann-Whitney was applied to estimate possible differences between median measurements of inner ear structures. Receiver operating characteristics (ROC) and Univariate logistics regression curves were constructed to identify the cutoff points of the highest specificity and accuracy for the diagnosis of CSNHL. For all the analyses, a 5% significance level and a 95% test power were considered (Statistic 10.0., StatSoft Inc., Tulsa, OK, USA).

## Results

The study included 214 patients undergoing investigation of acquired sensorineural hearing loss (ASNHL group) (n = 164), who formed the control group, and patients with congenital sensorineural hearing loss (CSNHL group) (n = 50).

The 214 patients averaged 45 years of age (IQR = 35–52), with no difference regarding gender ( *p* = 0.15).

A comparison of the inner ear measurements between groups showed that in the CSNHL group, median CBI measurements were 0.48 mm smaller ( *p* < 0.001), CNA measurements were 0.10 mm smaller ( *p* < 0.001), and CH measurements were 0.15 mm smaller ( *p* < 0.001). For VA and CW, the measurements were similar between the ASNHL group and CSNHL group (0.70 *vs* 0.72, *p* = 0.19 e 7.20 *vs* 7.15 *p* = 0.23) ( Table 1 ).

**Table 1:** Comparison of measurements according to group with acquired sensorineural hearing loss and congenital sensorineural hearing loss

Measurements	ASNHL (n = 164)	CSNHL (n = 50)	*p*
CBI	4.00 (3.75–4.25)	3.52 (2.25–4.00)	**< 0.001**
VA	0.70 (0.62–0.75)	0.72 (0.60–1.10)	0.19
CNA	1.60 (1.50–1.70)	1.50 (1.00–1.60)	**< 0.001**
CH	3.65 (3.50–3.77)	3.50 (3.05–3.80)	**< 0.001**
CW	7.20 (7.05–7.37)	7.15 (7.05–7.30)	0.23

Abbreviations: ASNHL, acquired sensorineural hearing loss; CBI, central bony island; CH, cochlea height; CAN, cochlear nerve aperture; CSNHL, congenital sensorineural hearing loss; CW, cochlea width; VA, vestibular aqueduct.

Note: Mann-Whitney test.

**Fig. 2:**
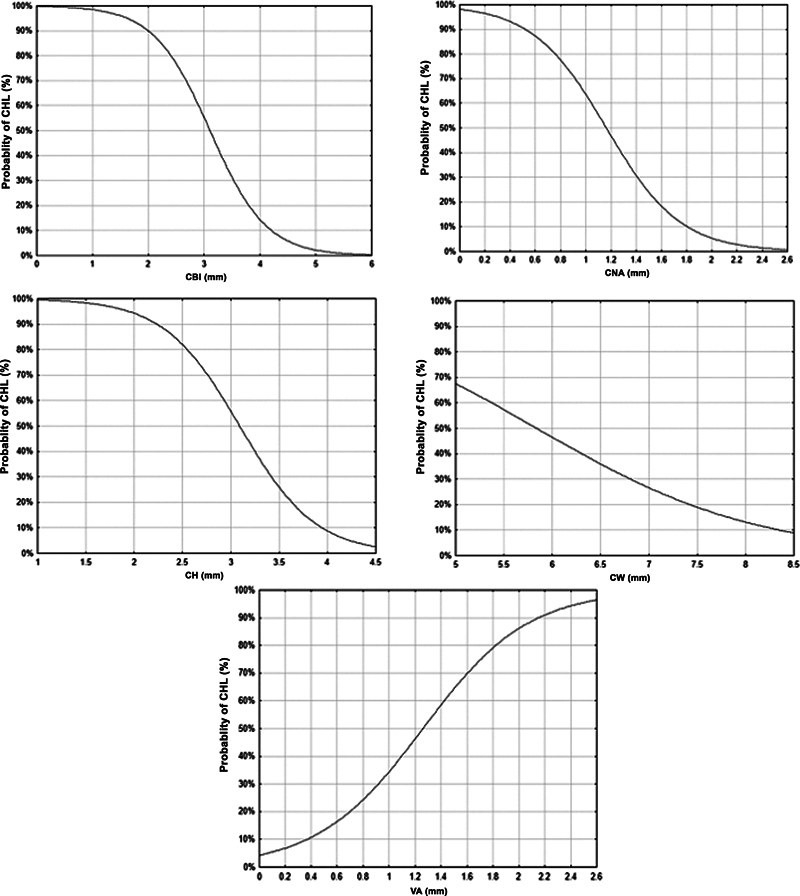
Probability of congenital sensorineural hearing loss according to inner ear measurements. CBI = Central bony island; OCN = Opening of the cochlear nerve; CH = Cochlea height; CW = Cochlea width; VA = Vestibular aqueduct. Logistic regression model: *p* < 0,001.

In the univariate logistic regression analysis, performed to estimate the probability of CSNHL according to inner ear measurements, it was observed that the smaller the measurement of the CBI, the higher the probability of CSNHL. Thus, with measurements smaller than 3.6 mm, the probability of CSNHL was approximately 30%, rising to 55% with a measurement of 3 mm, and from 90 to 100% with measurements smaller than 2 mm ( *p* < 0.001) ( Fig. 2 ).

It was also observed that the smaller the measurement of the CNA, the higher the probability of CSNHL. Thus, with measurements smaller than 1.4 mm, the probability of CSNHL was approximately 30%, rising to approximately 50% with a measurement of 1.2 mm, and from 85 to 100% with measurements smaller than 0.6 mm ( *p* < 0.001) ( Fig. 2 ).

Similarly, it was observed that the smaller the measurement of the CH, the higher the probability of CSNHL. Thus, with measurements smaller than 3.4 mm, the probability of CSNHL was approximately 30%, rising to approximately 60% with a measurement of 3.0 mm, and from 80 to 90% with measurements smaller than 2.5 mm ( *p* < 0.001) ( Fig. 2 ).

For the CW measurement, with measurements smaller than 7.0 mm, the probability of CSNHL was approximately 30%, rising to approximately 70% with a measurement of 5.0 mm ( *p* = 0.04) ( Fig. 2 ).

The probability of CSNHL increases with the diameter of the VA. Thus, with measurements smaller than 0.9 mm, the probability of CSNHL was approximately 30%, rising to 60% with a measurement of 1.4 mm, and from 90 to 100% with measurements greater than 2.2 mm ( *p* < 0.001) ( Fig. 2 ).

The measurements showed low sensitivity (18–56%), but high specificity (88.4–98.8%) and accuracy (73.8–82.7%) for the suggested cutoff points ( Table 2 ).

**Table 2:** Values of sensitivity, specificity, cutoff points and area under the ROC curve of inner ear measurements for the diagnosis of congenital sensorineural hearing loss

Measurements	Sensitivity	Specificity	Accuracy	Cutoff point	AUC
CBI	56.0	88.4	80.8	3.65	0.74
CNA	34.0	97.6	82.7	1.4	0.67
CH	34.0	96.3	81.8	3.4	0.63
CW	18.0	90.8	73.8	7.1	0.56
VA	30.0	98.8	82.7	0.9	0.56

Abbreviations: AUC, area under the curve; CBI, central bony island; CH, cochlea height; CAN, cochlear nerve aperture; CW, cochlea width; VA, vestibular aqueduct *.*

Note: ROC curve.

## Discussion

In the current sample, it was observed that patients with CSNHL had lower values in the measurement of CBI, CNA, and CW. The cutoff points that should cause concern for the diagnosis of CSNHL and early intervention include 3.6 mm for the CBI measurement, 1.4 mm for the CNA, 3.4 mm for the CH, 7.0 mm for the CW, and 0.9 mm for the VA.

It is estimated that in more than half of SNHL cases, etiology is associated with the genetic origin of recessive inheritance in 75 to 80% of the cases. In 15 to 20%, the inheritance is dominant, and in 1 to 2%, it is linked to the X chromosome. Approximately 30% of the hereditary forms are syndromic, with more than 300 described conditions, and the remaining 70% are non-syndromic. Of the non-genetic conditions, an environmental cause is identified in about half of the cases, and in the other half, it is considered idiopathic. 2

The discussion related to inner ear diseases and malformations has advanced in the last few decades. Imaging, CT scan, or magnetic resonance imaging (MRI) exams have been recommended as essential tools to identify the etiology of HL and abnormalities that may be predictive for its diagnosis. 2 In the CT scan of the inner ear in patients with CSNHL, malformations have been observed in 7 to 20% of the cases. 2

The use of CT scan to assess CSNHL is important for its investigation and surgical planning. 3 The CT scan's objective is to assist radiologists in obtaining a more accurate diagnosis of the underlying etiology and assist surgical planning. 4 5

Although both techniques (CT scan and MRI) may be used to assess malformations of the inner ear, the CT scan is better for abnormalities of the middle or outer ear. In contrast, for the inner ear (membranous labyrinth and nerves of the internal auditory canal), preference should be given to MRI. 6

Considering that the size of the inner ear remains the same since birth, adults and children were included in the present sample with the goal of assessing the anatomical differences in the structures of the middle ear in patients with congenital and acquired SNHL. However, it must be acknowledged that the linear measurements of inner ear structures, both by CT scan and MRI, significantly limit the ability to assist in the diagnosis of dysplasia. For this reason, the development of two-dimensional or three-dimensional reconstruction techniques, can improve calculations of areas or volumes. 7 8 9

The interpretation of the CT scan is highly dependent on experience, and visual inspection is sensitive for the diagnosis of more severe malformations, which represent only 1% of radiographic abnormalities found in patients with SNHL, and many minor abnormalities are not diagnosed. Thus, objective radiological measures reduce dependence on technical experience, increase sensitivity in minor abnormalities, and reduce diagnostic errors. 10

In the present sample, five measurements of inner ear structures were verified in patients with CSNHL.

### Central Bony Island

Regarding the CBI measurement, it was observed that CSNHL determined a decrease of about 0.48 mm. Acquired sensorineural hearing loss patients presented a measurement of approximately 4 mm, while patients with CSNHL presented approximately 3.5 mm. Thus, a decrease in the measurement of CBI was associated with a higher probability of CSNHL, with a cutoff point of 3.6 mm, from which the probability of CSNHL was greater than 30% with high specificity and accuracy (88.4–80.8%). Some authors found a cutoff point of 2.8 mm, with measurements in the control group ranging from 2.8 to 4.8 mm and from 1.5 to 4.8 mm in the group with CSNHL. 11

Another study found that 62.3% (48/77) of the temporal bones with congenital anomalies had abnormalities in the vestibular system. 12 Of the 117 individuals' vestibular abnormalities, 73 (62.4%) were in the semicircular canals. Among these, the lateral semicircular canal (LSCC) was the most affected, possibly explained by the later development of this anatomical structure. However, the impression remains that the VA, not the semicircular canals, is the most affected structure in patients with inner ear anomalies. 12

Subtle abnormalities of the inner ear, such as cochlear hypoplasia and dysplasia of semicircular canals, which are responsible for most inner ear malformations associated with SNHL, are generally not seen in the temporal bone CT scan due to inexperience and lack of normative data to assist diagnosis. 13 14 The routine measurement of cochlear height and CBI of the lateral semicircular canal, together with a visual inspection of CT images, increases the recognition of common inner ear malformations. 15 Other authors suggested that routine measurement of height and the cochlear CBI of the LSCC can help in the recognition of the most common inner ear malformations. 16

### Vestibular Aqueduct

In the present study, VA measurements were of approximately 0.70 mm in ASNHL, rising by about 0.02 mm, on average, with CSNHL. The increase in VA measurement was associated with a higher probability of CSNHL, with a cutoff point of 0.9 mm, from which the probability of CSNHL was greater than 30%, with high specificity and accuracy (98.8% and 82.7%).

The enlarged vestibular aqueduct (EVA) is the most common malformation in children with CSNHL, which is defined when greater than 1.5 mm. 2 17 18 19 20 21

When assessing the EVA syndrome, some authors performed expanded radiographic evaluations of 50 patients with VA greater than 1.5 mm. The presence of other inner ear abnormalities was identified in 60% of this population. 22

Some authors confirmed that modiolus abnormality was found in all 93 patients with EVA, suggesting that this enlargement is not an isolated problem. 23

On the other hand, the assessment of SNHL in children was also examined in another study with 114 participants with syndromic SNHL or not. Of the 97 patients who underwent radiological studies, 38 of them (39%) presented abnormalities. Isolated inner ear malformations, especially EVA, were twice as common as multiple abnormalities, followed by LSCC dysplasia, cochlear dysplasia, and small internal auditory canal. These seem to be, in fact, the most important measurements for radiological diagnosis. 24

### Cochlear Nerve Aperture

Two studies found significantly smaller width and height of the cochlear nerve canal in a group of 33 children with profound SNHL, previously assessed as normal in a CT scan, compared to 50 children with normal hearing. The hypoplastic bony canal of the cochlear nerve in patients with SNHL may indicate previously unrecognized embryological malformation of the cochlear nerve. Thus, they observed that the length and width of the bony canal for the cochlear nerve were significantly shorter in patients with SNHL and concluded that hypoplasia of the bony canal might indicate a previously unrecognizable embryological malformation of the cochlear nerve. 6 25

Some authors observed measurements of 1.91 ± 0.27 mm in patients without SNHL and 0.99 ± 0.37 mm in patients with SNHL. 26 In another research, authors have pointed out normal measurements ranging from 1.4 to 3.00 mm. 2 The absence or reduction of this canal is observed in 12 to 18% of CSNHL and measurements below 1.4 or 1.5 mm indicate abnormality. 26 27

In the present study, it was observed that CSNHL determined a decrease in CNA, of approximately 0.1 mm. Thus, the decrease in CNA was associated with a higher probability of CSNHL, with a cutoff point of 1.4 mm, from which the probability of hearing loss was greater than 30%, with high specificity and accuracy (97.6% and 82.7%). Stjernholm and Muren found a similar cutoff point, of 1.4 mm, to indicate the probability of abnormality in the cochlear nerve. 27

### Cochlea Height

The CH was, on average, 3.65 mm in the ASNHL, decreasing by about 0.25 mm with the CSNHL. Thus, the decrease in CH was associated with the higher probability of CSNHL, with a cutoff point of 3.4 mm, from which the probability of CSNHL was greater than 30%, with high specificity and accuracy (96.3% and 81.8%).

Some authors carried out a study to standardize measurements of the inner ear in children with CSNHL with minor malformations and roughly normal CT scan. Measurements were made of 45 ears with SNHL, with normal CT scan, and 45 in the control group. Children with clearly abnormal CT scans or other diseases such as cytomegalovirus, measles, or other diagnostics were excluded. Measurements of CBI and lumen of the semicircular canals, height, and CW were performed. The authors found significant differences in CH, CBI of the superior semicircular canal (SSCC), and LSCC, with the height being significantly lower in the group with SNHL (4.79 vs. 4.46 mm). Central bony island of the SSCC (4.79 vs. 5.06) and LSCC (3.41 vs. 3.6 mm) measurements were also significantly lower in the SNHL group. 28

### Cochlea Width

There was no significant alteration of the CW measurement. The measurement was, on average, 7.2 mm in the ASNHL and remained around this in the CSNHL. A decrease greater than 7.0 mm was associated with a probability of CSNHL greater than 30% with specificity of 90.8% and an accuracy of 73.8%.

Another paper correlated the cochlear measurements and CSNHL. They found that the cochlea width was significantly smaller in the CSNHL group and a size smaller than 5,4 mm was highly suggestive of hearing loss. 29

Würfel et al *.* specifically discussed how to determine the length of the cochlea from cone-beam CT scan in candidates for a cochlear implant. Using a broad database with 218 temporal bones exams, they observed that the length differed between men and women but was not different according to age or sides. The average cochlear length was 37.6 mm (SD: ± 1.93 mm), with a median of 37.6 mm (32–43.5 mm). In addition, the authors found that the cochlea size presented a normal distribution in the sample. 30

The non-normality in the distribution of measurements of width and CH indicate the need for further studies, given the importance of the size of the structures for the preoperative planning of cochlear implantation, as well as length identification techniques. 29 30 31 32

## Conclusion

Congenital sensorineural hearing loss determined a decrease in CBI, CNA, and CW. The predictive cutoff points for CSNHL were 3.6 mm for the CBI measurement, 1.4 mm for the CNA, 3.4 mm for the CH, 7.0 mm for the CW, and 0.9 mm for the VA. Thus, these measures, at the cutoff points indicated, should make us aware of the diagnosis of congenital disease and provide the opportunity for early intervention to avoid delay in the child's overall development.
